# Transcatheter Closure of Apical Septal Rupture With Third Cavity Formation After Surgical Repair

**DOI:** 10.1016/j.jaccas.2026.110217

**Published:** 2026-10-07

**Authors:** Saqlain Anwar, Muhammad Ammar Nadeem, Hritvik Jain, Nadeem Sadiq, Areeba Shahid, Aizaz Anwar Khalid, Abhishek Kumar, Yasar Sattar

**Affiliations:** a Department of Internal Medicine, PNS Shifa Hospital, Karachi, Pakistan; b Department of Internal Medicine, Allegheny General Hospital, Pittsburgh, Pennsylvania, USA; c Department of Pediatric Cardiology, PNS Shifa Hospital Karachi, Pakistan; d Department of Internal Medicine, Jinnah Sindh Medical University, Karachi, Pakistan; e Peshawar Medical College, Peshawar, Pakistan; f Internal Medicine, Wright State University, Dayton, Ohio, USA; g Texas Tech University, Texas, USA; h West Virginia University, Camden Clark Medical Center, Parkersburg, West Virginia, USA; i Interventional and Endovascular Cardiologist, Appalachian Regional Hospital, Beckley, West Virginia, USA

**Keywords:** case report, percutaneous intervention, reoperation alternative, ventricular septal defect, ventricular septal rupture

## Abstract

**Background:**

Apical septal rupture following surgical closure of a ventricular septal defect is a rare but life-threatening complication. Reoperation in such cases may be high risk, making transcatheter closure a viable and less invasive alternative.

**Case Summary:**

We report an 8-month-old male infant who presented with cardiac failure with Ross class-III and severe respiratory distress. Clinical examination and imaging revealed an apical septal tear with formation of a third cavity extending toward the right ventricle and compromising right ventricular and left ventricular function. The patient underwent successful percutaneous device closure of the defect with complete recovery and no complications.

**Discussion:**

This case highlights the safety and efficacy of transcatheter closure in complex postsurgical ventricular septal defects. The minimally invasive nature of this approach makes it preferable in high-risk surgical candidates. Further studies are warranted to standardize outcomes for such interventions.

**Take-Home Message:**

Transcatheter closure is a safe, effective alternative in postsurgical congenital heart defects where reoperation poses a significant risk.

## Visual Summary

**Figure:**
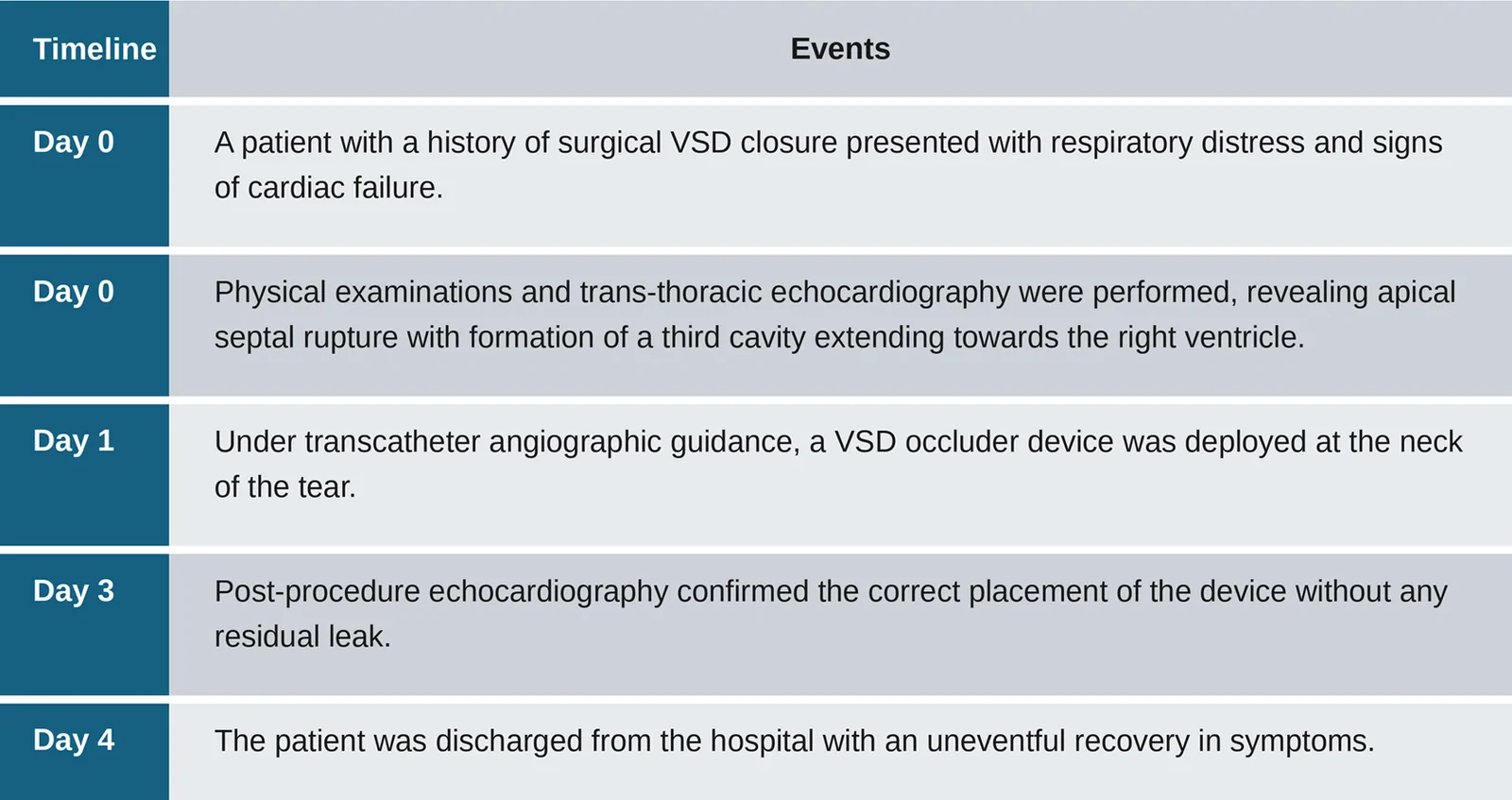

## Case Presentation

### History of presentation

An 8-month-old male infant, who had undergone ventricular septal defect (VSD) patch closure at 6 months of age, came into the emergency department with respiratory distress and signs of heart failure. The parents mentioned that over the past 2 weeks, the baby had been having trouble feeding, irritability, and sweating. They also noticed reduced activity, lethargy, and poor weight gain since the VSD closure 2 months ago. Based on these symptoms, the infant's heart failure was classified as class III, according to the “Modified Ross Classification for Pediatric Heart Failure.”^1^

On examination, the baby was breathing fast, with nasal flaring and subcostal retractions. Growth parameters showed weight and length below the 10th percentile. Vital signs showed a heart rate of 165 beats/min, respiratory rate of 50 breaths/min, 108/65 mm Hg blood pressure with a narrow pulse pressure, oxygen saturation of 92% on room air, and a capillary refill time of 3.5 seconds. A harsh, grade III/VI holosystolic murmur was heard along the left lower sternal border, suggesting a residual or recurrent VSD. Lung examination revealed bilateral coarse breath sounds, and there was bilateral fine crepitus over the lung bases. The rest of the examination was unremarkable.

### Past medical and surgical history

The infant had undergone surgical patch closure of a large outlet muscular VSD at 6 months of age, with episodes of repeated acute respiratory infection since 2 months of age. No other significant past medical issues were reported.

### Brief description of surgical procedure

After establishing cardiopulmonary bypass (CPB), a cardioplegia cannula was placed in the aortic root. The aortic cross-clamp was then applied, and antegrade cold-blood Del Nido cardioplegia (30 mL/kg) was delivered to achieve adequate diastolic cardiac arrest and to ensure coronary protection. A vent was placed in the left heart via the right superior pulmonary vein. Caval tapes were then snared. The patent ductus arteriosus was ligated as per routine.

A right atriotomy incision was made. Attention was turned to visualizing the VSD. It was a large-sized, high muscular type of VSD. Working through the tricuspid valve, this VSD was closed with a Dacron patch that was secured with running 5-0 Prolene suture inferiorly and superiorly, with interrupted pledgeted 5-0 Prolene sutures passed through the annulus of the tricuspid valve superiorly. The VSD appeared adequately closed. The tricuspid valve was tested and appeared competent. The right atriotomy was closed in two layers.

Perfusion data: CPB time was 48 minutes, and aortic cross-clamp time was 31 minutes.

The patient was discharged home on the fifth day after surgery.

### Investigations

Laboratory tests revealed hemoglobin of 11 g/dL, hematocrit of 34%, and platelets of 255,000/μL, with normal kidney and liver function tests. His chest x-ray film was unremarkable. Transthoracic echocardiography revealed an apical septal tear with an aneurysmal pouch protruding toward the right ventricle and causing dyskinesia of the septum. There was severe left ventricular (LV) dysfunction with ejection fraction 35%, tricuspid annular plane systolic excursion 10 mm, and 7-8 mm of neck of blind sac of apical part of septum with formation of a third cavity extending toward the right ventricle (Figures 1A and 1B, Video 1).

**Figure 1:**
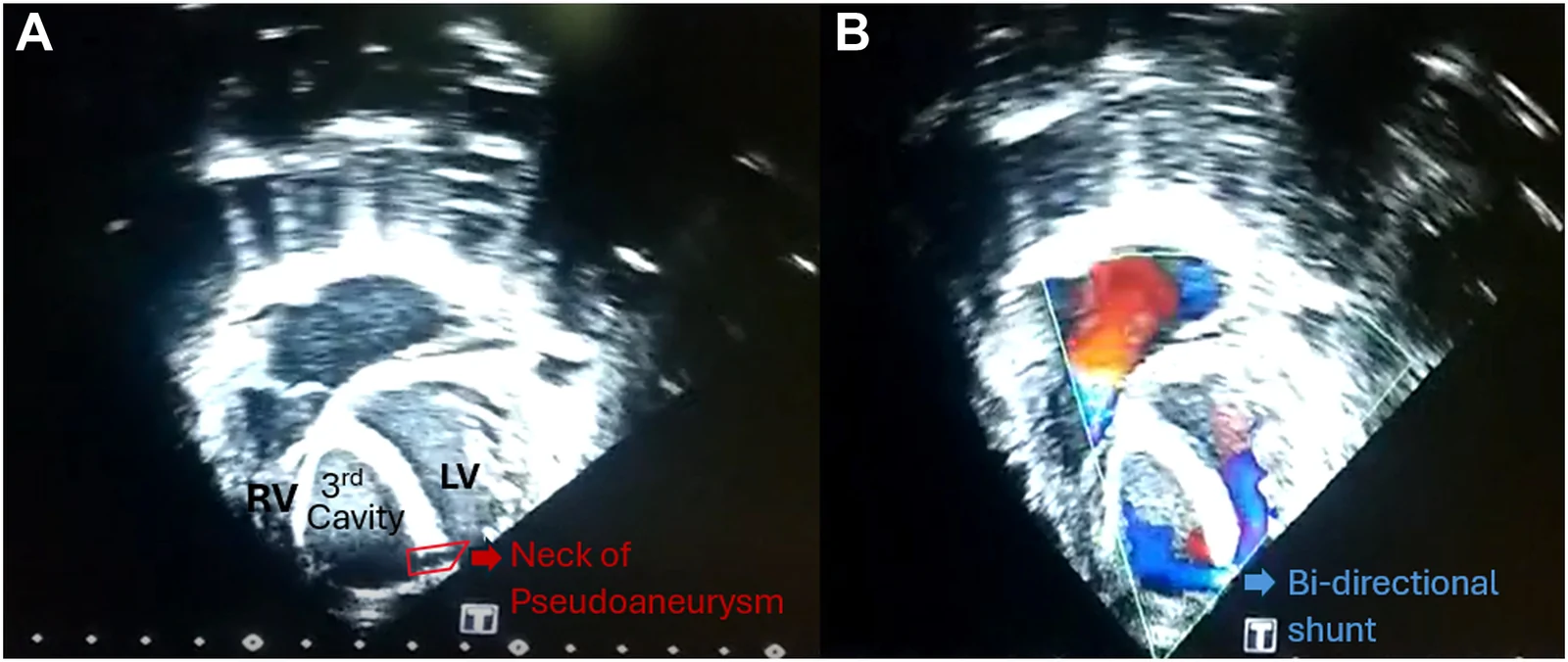
Transthoracic Echocardiography in the Apical 4-Chamber View (A) Two-dimensional image showing the pseudoaneurysm presenting as a third cavity (“third cavity”) located between the right ventricle (RV) and left ventricle (LV); the red arrow indicates the neck of the pseudoaneurysm at the site of the previous surgical ventricular septal defect closure. (B) Color Doppler imaging demonstrating bidirectional shunting through the neck of the pseudoaneurysm (arrow).

### Management

The case was reviewed in a multidisciplinary team meeting. Given the young age of the patient and the significant risk associated with reoperation, a percutaneous transcatheter approach aiming at correcting the defect was opted.

The procedure was performed under general anesthesia with informed parental consent. LV angiography demonstrated a large apical tear (7-8 mm) leading to a blind sac with normal coronary artery anatomy (Figures 2A and 2B, Video 2). A 14/12 mm Konar-MF (Lifetech Scientific) was selected to close the defect. This multifunctional occluder has a unique design with an asymmetrically truncated disc connected to an opposing disc, resulting in a soft, lightweight profile that can be delivered through a small sheath. These characteristics may minimize trauma to adjacent tissues in small infants while providing stable anchorage within the defect. A 6-F sheath was advanced into the blind sac (Figure 2C). Postdeployment angiography confirmed correct device placement with no residual leak (Figure 2D, Video 3).

**Figure 2:**
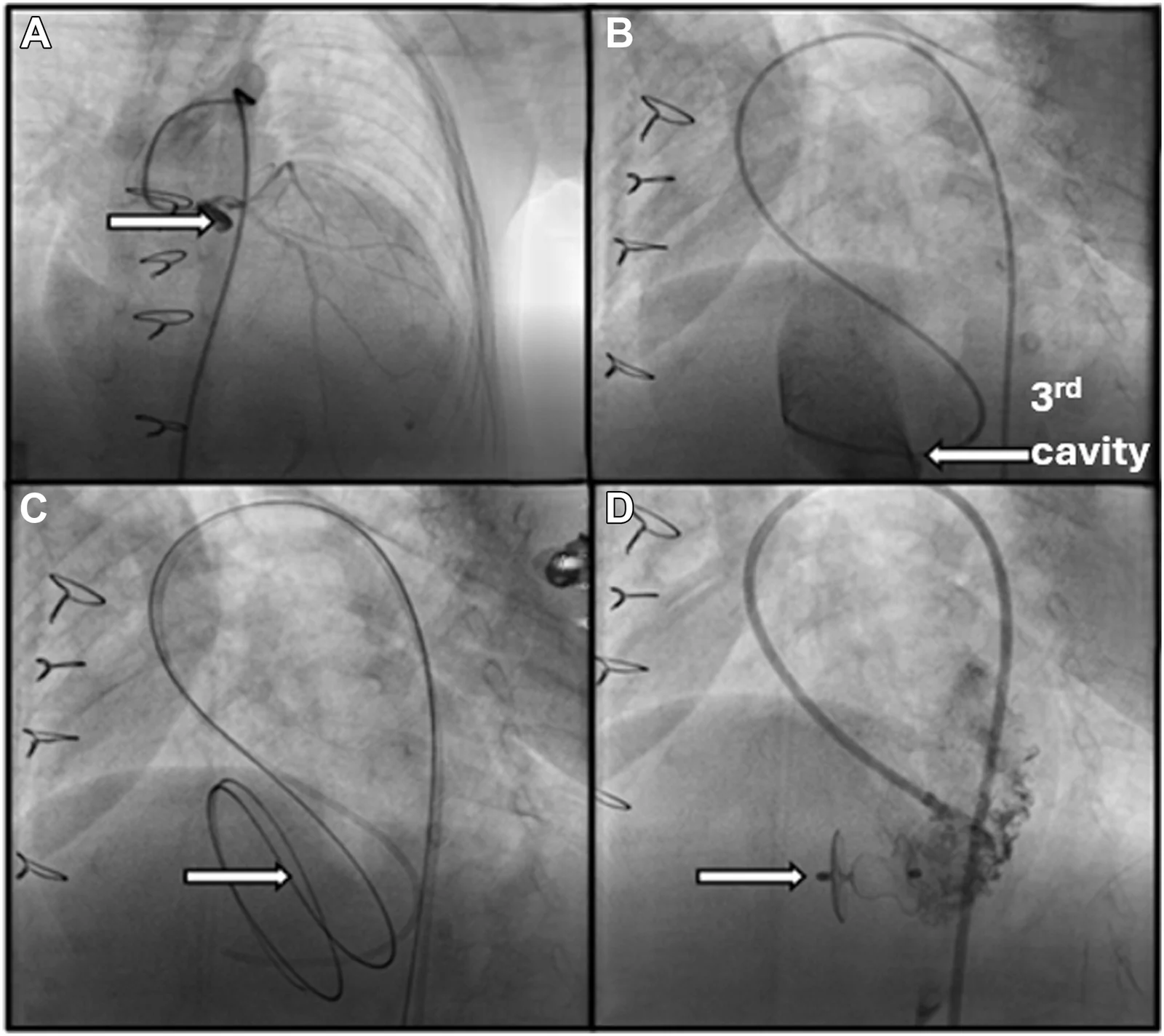
Transcatheter Closure of the Apical Septal Rupture on Angiography (A) Cardiac angiography showing the left coronary artery (arrow). (B) The catheter is in the blind pouch (third cavity) extending toward the right ventricle as labeled by the arrow. (C) The Terumo wire and the catheter (arrow) are positioned in the cavity. (D) Postprocedure left ventricle angiogram showing a well-placed device (arrow).

### Outcome and follow-up

The infant had an uneventful recovery and was discharged 3 days after the procedure. At a follow-up visit on day 7 and 30, the patient remained asymptomatic, with no evidence of cardiac failure or residual leak on echocardiography. The patient continues to thrive and stay complication-free under regular follow-up. At 3-year follow-up, echocardiography showed complete resolution of the third cavity with normal biventricular function (Video 4).

### Discussion

VSD is among the most common congenital heart conditions. Although surgical repair is the gold standard method, challenges such as residual VSDs and septal rupture can still occur.^2^ In this case, the infant developed an apical septal rupture following VSD patch closure, requiring urgent intervention. Because of the high risks involved in reoperation, we chose transcatheter closure as a more secure and practical option.

The exact underlying cause of the pseudoaneurysm could not be clearly delineated, and it remains idiopathic. There are several proposed mechanisms for postsurgical apical ruptures, including suture line dehiscence, patch margin breakdown, surgical trauma, localized ischemia, and infections or inflammation, which can weaken the surrounding tissue.^3^ The most probable mechanism behind the formation of a pseudoaneurysm in this case was hypothesized to be because of inadvertent retractor injury at the apical part of the septum that became weak and resulted in thinning of the septum. The sustained LV pressure on the weakened area of the septum resulted in the formation of a progressive blind pouch, which compromised the heart's function. These kinds of unclear precipitating factors for postoperative complications highlight the importance of close echocardiographic follow-up to monitor the cardiac structural integrity following VSD closure. The surgical intervention typically involves patch closure under CPB. The surgical procedure has a few documented complications, such as complete atrioventricular block, the possibility of reoperation, bleeding, and, in rare cases, neurological complications.^2^ Transcatheter closure is a common and effective option, especially for high-risk cases where a second surgical intervention may not be ideal.^4^ Prospective studies have shown the transcatheter route to be comparably successful, with closure rates ranging from 90% to 100%.^5^^,^^6^ Further developments in occluder devices, like the Amplatzer (Abbott) and other modern designs, have made deployment very precise.^7^ As in this patient, the septal rent was at the apex, and for the surgeon its far away to patch the site, so it usually needs a transapical approach that can result in long-term rhythm problems because of scar formation at the incision site. Although transcatheter closure is beneficial, it carries certain risks, including device embolization, conduction issues, interference with heart valves, and residual leaks.^9^ In this case, we used detailed angiographic and echocardiographic measures to position the device to mitigate these risks carefully. Fortunately, no conduction problems were found during follow-up, showing the safety of this procedure. One common concern usually associated with transcatheter closure is the risk of complete atrioventricular block, with reported incidence rates ranging from 0% to 5.7%. Given the apical location of the pseudoaneurysm, other potential complications associated with device closure include apical free-wall rupture, device-related thrombosis, systemic thromboembolism, residual aneurysm formation, or device erosion.

However, studies have shown that the use of refined implantation techniques and careful patient selection has helped minimize this risk over time.^5^

Studies indicate that transcatheter closure has good long-term outcomes, as most patients show improvement in their symptoms and a stable defect closure.^10^ The current patient remained asymptomatic during follow-up, with no residual shunting or signs of cardiac dysfunction, reinforcing the durability of this approach. Still, regular monitoring is important to detect any late-onset conduction problems or device-related issues.^2^

Existing literature suggests that additional devices are sometimes needed during the initial procedure or shortly after in patients undergoing transcatheter closure because of residual shunts. Over time, significant residual shunts often decrease, and in some cases, they resolve on their own.^8^ Multiple studies have indicated that transcatheter closure is associated with lower reoperation rates than surgical repair. Research shows that while some cases might need further interventions, transcatheter closure has typically reduced the need for repeat surgeries, making it a preferred option for selected patients.^7^^,^^8^

## Conclusions

This case highlights the success of transcatheter closure for managing apical ventricular rupture after VSD patch repair in an infant. It offers a practical and less invasive option for patients at high surgical risk with favorable clinical results. Continued advancements in device technology call for further research and long-term follow-up to enhance patient selection and improve outcomes.

**Visual Summary:**
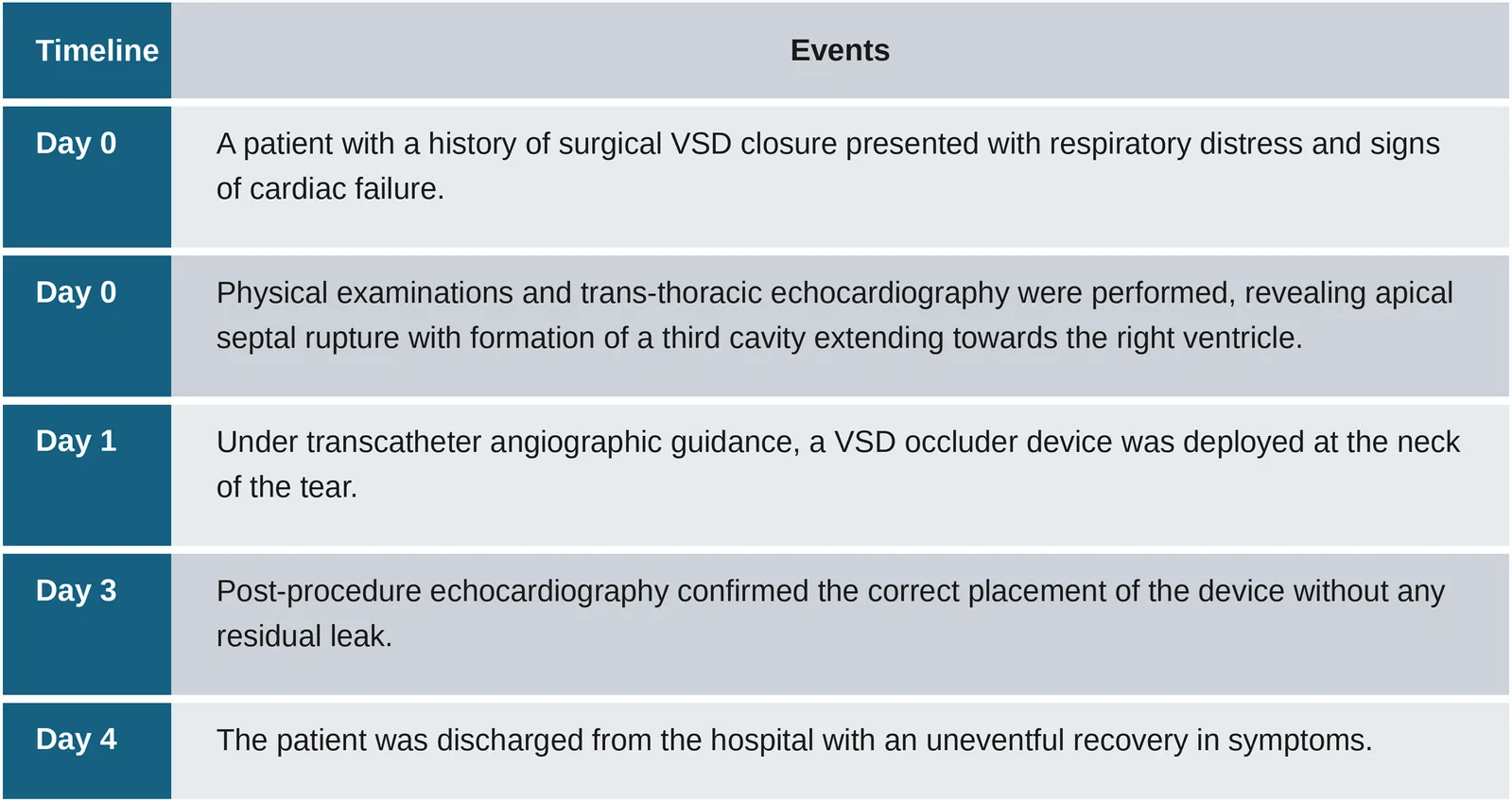
Timeline of the Case VSD = ventricular septal defect.

## Funding Support and Authors Disclosures

The authors have reported that they have no relationships relevant to the contents of this paper to disclose.

**Take Home Messages**

• Apical ventricular septal rupture after surgical repair is a very rare and potentially life-threatening emergency, which poses a significant risk to patients.
• High-risk patients, especially infants, presenting with heart failure after a surgical ventricular septal defect patch closure, warrant immediate multimodality imaging for the early diagnosis and management.
• In patients with such complications from a recent surgery, reoperation poses a significant risk; transcatheter management by deploying a Konar-MF Occluder device offers a safe and effective alternative.

## Appendix

Video 1 Transthoracic Echocardiography Showing the Apical Septal Rupture With an Aneurysmal Pouch (Third Cavity) Extending Toward the Right Ventricle and Bidirectional Shunting Across the Neck of the Pseudoaneurysm

Video 2 Cardiac Angiography Showing the Catheter Positioned in the Blind Pouch (Third Cavity) Extending Toward the Right Ventricle, Before Device Deployment

Video 3 Postdeployment Angiography Showing Correct Device Placement With no Residual Leak A 14/12-mm Konar-MF occluder, a self-expanding double-disc VSD occlusion device (Lifetech Scientific), was deployed to close the defect.

Video 4 Transthoracic Echocardiography at 3-Year Follow-Up Showing Complete Resolution of the Third Cavity The device is well seated at the apical septum with no residual shunt on color Doppler. Biventricular size and systolic function are normal.
